# The MLKL kinase-like domain dimerization is an indispensable step of mammalian MLKL activation in necroptosis signaling

**DOI:** 10.1038/s41419-021-03859-6

**Published:** 2021-06-22

**Authors:** Yu Zhang, Jia Liu, Dandan Yu, Xinxin Zhu, Xiaoyan Liu, Jun Liao, Sheng Li, Huayi Wang

**Affiliations:** 1 School of Life Science and Technology, ShanghaiTech University, 393 Middle Huaxia Road, Pudong, Shanghai, 201210 China; 2 State Key Laboratory of Cell Biology, CAS Center for Excellence in Molecular Cell Science, Shanghai Institute of Biochemistry and Cell Biology, Chinese Academy of Sciences, University of Chinese Academy of Sciences, Shanghai, 200031 China; 3 University of Chinese Academy of Sciences, Beijing, 100049 China; 4 Shanghai Institute for Advanced Immunochemical Studies, ShanghaiTech University, 393 Middle Huaxia Road, Pudong, Shanghai, 201210 China

**Keywords:** Necroptosis, X-ray crystallography

## Abstract

MLKL phosphorylation by RIP3 is the commitment step of necroptosis execution, which could induce MLKL activation featured as MLKL monomer-oligomer transition. Here, we reported that the dimerization of the MLKL kinase-like domain was the direct consequence of RIP3 triggered MLKL-phosphorylation. Two inter-dimer interfaces were found in the crystal structure of human MLKL. Mutations destroying both interfaces could prevent RIP3-induced MLKL oligomerization and necroptosis efficiently. Moreover, we confirmed MLKL self-assembly by the internal coiled-coil region is necessary for MLKL oligomerization and function. The mutations disrupting coiled-coil self-assembly repressed necroptosis, but it did not prevent RIP3-induced dimerization of the MLKL kinase-like domain. So that, MLKL activation is a sequential process, which begins with kinase-like domain dimerization, and followed by internal coiled-coil region self-assembly to form a proper MLKL oligomer. Besides human MLKL, structural and functional analysis showed the kinase-like domain dimerization was conserved among mammalian species, suggesting it is a general step of the RIP3-induced MLKL activation process.

## Introduction

Mixed Lineage Kinase domain-like protein (MLKL) is the executioner of necroptosis, a type of programmed cell necrosis. The MLKL caused necrosis participates in the serval physiological events including immune response against pathogens, aging-associated deterioration of the male reproductive system, and satellite cells (muscle stem cells) proliferation in myofiber regeneration^1–4^. MLKL protein is compromised a N-terminal four-helix bundle (4HB) region taking the charge of membrane disruption, the direct cause of cell necrosis, an internal coiled-coil (CC) region, and a C-terminal kinase-like domain (KLD)^5,6^. Usually, necroptosis signaling is mediated by two specific kinases, receptor-interacting protein kinase-1 (RIP1) and RIP3, which are sequentially activated by various extracellular cytokines such as the tumor necrosis factor (TNF)^2,7–11^. While the activation of MLKL is mainly regulated by RIP3-mediated phosphorylation at threonine 357 and serine 358 of human MLKL (or serine 345 of mouse MLKL) in the activation loop of the kinase-like domain^12^. Since the kinase-like domain of MLKL is catalytically defective, the phosphorylation on the activation loop does not boost the kinase-activity but relieves the auto-inhibitory monomer state of MLKL to promote its oligomerization through the coiled-coil domain-mediated self-assembly^12–14^. The oligomerized MLKL proteins translocate to plasma membranes, where the peptides corresponding to the four-helix bundle region insert into the membrane and form channels or pores, disrupting plasma membrane integrity and causing cell necrosis eventually^14–17^. Besides phosphorylation mediated by RIP3, other modifications on the MLKL kinase-like domain were reported to regulate MLKL function. One is Tyr376 of human MLKL, which is phosphorylated by membrane-associated TAM kinases to promote further oligomerization of membrane-binding MLKL and facilitate necroptosis execution^18^. Another is serine 441 of mouse MLKL. This phosphorylation site is mediated by unknown kinases and function to promote myelin breakdown in Schwann cells, a MLKL-dependent but RIP3-independent process in axon regeneration^19^. Therefore, the kinase-like domain of MLKL is considered as integrator for upstream signals to regulated MLKL activation.

In normal conditions, the kinase-like domain maintain the auto-inhibitory state of MLKL, which is revealed by the crystal structure of the full-length mouse MLKL (PDB code: 4BTF)^5^. In this structure, the internal coiled-coil region of MLKL is restricted by both the N-terminal four-helix bundle and C-terminal kinase-like domain. A series of electrostatic interactions including R34-E135, R30-D139, and K26-E142 are present between residues in helix α2 of the four-helix bundle and helix α5 of the coil-coiled region in this structure^5^. These interactions are also present in the solution structure of the N-terminal part of human MLKL (PDB code: 2MSV)^20^. Mutation disrupting these interaction such as D139V in mouse MLKL and K26E/R30E in human MLKL could induce MLKL activation which elevates the in vitro liposome leakage efficiency of the MLKL proteins and cause necroptosis bypass the RIP3 regulation^20,21^. It suggested the four-helix bundle is directly inhibited by the monomeric coiled-coil region. Besides these mutations on MLKL four-helix bundle and coiled-coil region, there are pseudoactive sites on MLKL kinase-like domain including phosphomimic S345D mutation in mouse MLKL (or T357E/S358D mutations in human MLKL) and mutations disrupting an unusual salt-bridge K219–Q343 present in mouse MLKL structure. Both of the phosphomimic mutants and mutants changing the sites of K219/Q343 in mouse MLKL (or conserved sites K230/Q356 in human MLKL) induce MLKL oligomerization and activation which causes RIP3-independent necroptosis^5,14,18^. While mutations on the coiled-coil region of MLKL, disrupting their self-interaction block RIP3-induced MLKL oligomerization and necroptosis^13^. Therefore, MLKL activation by the upstream signal is initiated by changing the kinase-like domain, which induces oligomerization of the coiled-coil region. But the how the monomer to oligomer transition of the coiled-coil region controlled by kinase-like domain is still uncovered. Here, we report the phosphorylation by RIP3 can induce the dimerization of the kinase-like domain of MLKL, which is the critical step to induce the following oligomerization and activation of full-length proteins. The crystal structure reveals two binding interfaces in MLKL kinase-like domain dimer. The mutations to disrupt interactions of both interfaces could prevent MLKL dependent necroptosis efficiently. Besides that, we proposed sequential model of MLKL processing in necroptosis execution, and the function of MLKL kinase-like domain dimerization was to initiated oligomerization of MLKL internal coiled-coil region. It is confirmed by the mutations disrupting internal coiled-coil self-assembly, which repressed MLKL-dependent necroptosis downstream of RIP3-mediated MLKL KLD dimerization. More importantly, inconsistent with the previous species-divergent model of MLKL activation, the structural and functional analysis indicated kinase-like domain dimerization was the general step to commit RIP3-induced MLKL activation process among mammalian species.

## Materials and methods

### Reagents and antibodies

Recombinant TNF was purified in our lab. Necrostatin-1 (cat# HY-15760) is bought from MedChemExpress. Smac mimetic and ecrosulfonamide (NSA) compound was used as described previously^14^. z-VAD-fmk (cat# 1140) is bought from BioVision. Antibodies used for western blots were purchased from Abcam (anti-p-MLKL, cat# ab187091), MBL (anti-β-actin-HRP-DirecT, cat# PM053-7; anti-GAPDH, cat# M171-3), Sigma-Aldrich (anti-Flag, cat# F3165); Cell Signaling Technology (anti-rabbit IgG, cat# 7074).

### Molecular cloning

Standard PCR and cloning methods were used to clone the full-length or mutated cDNAs of MLKL into the lentiviral vector pCDH-CMV-MCS-EF1-copGFP (Addgene). Wild-type or mutated cDNAs of the full-length and kinase-like domain of MLKL were cloned into pFastBac-I vector (Life Technologies) for baculovirus expression systems or pCMV-N-flag vector(Clontech) for expression in mammalian cells. All plasmids were verified by standard DNA sequencing.

### Cell culture and stable cell lines

HT-29 human colon cancer cells, obtained from the cell bank of CAS (Shanghai). were cultured in Dulbecco’s modified Eagle’s medium/HIGH with L-glutamine, without sodium pyruvate (HyClone, cat# SH30003.02). All media were supplemented with 10% FBS (Gemini, cat# 900-108) and 100 units/ml penicillin/streptomycin (Invitrogen, cat# 15140163). All the cells were cultured at 37 °C with 5% CO_2_ and tested to be mycoplasma-negative by the standard PCR method. HT-29(*MLKL*-KO) cells were generated by using the CRISPR/Cas9-genome editing strategy and confirmed by PCR and western blot. The Cas9-target sites are as follows: human MLKL (5′-TACTCTTCAAGGACGTGAACAGG-3, 5′-TTCCCTTAGCAGAATCCACGGGG-3′). The stable cell lines were established by the lentiviral expression system. HEK293T cells were transfected with lentiviral vectors (pCDH-CMV-MCS-EF1-copGFP) and virus packing plasmids (psPAX2 and pMD2.G, Addgene cat#12259) by using EZ transfection reagents (Life iLab Biotech Co., Ltd, cat# AC04L092). The virus-containing medium was harvested 48 hr later and added to HT-29 MLKL KO cells as indicated, with 10 μg/ml polybrene.

### Chemical crosslinking assay

Crosslinker disuccinimidyl glutarate (Thermo Fisher Scientific, cat# 20593) was freshly made by dissolving in DMSO as a 20 mM stock solution. For proteins’ crosslinking, wild-type or mutant forms of MLKL recombinant kinase-like domain proteins were incubated with 500 μM DSG for 30 min at room temperature, along with DMSO as a negative control. The reaction was stopped by the addition of 100 mM Tris-HCl (pH 7.5) and incubated for 15 min at room temperature according to the user’s instruction. Then, samples were separated by SDS-PAGE and analyzed by Coomassie blue staining. For crosslinking in lysates, HEK293T or HEK293T-RIP3 cells plated in a 60 mm dish were transfected with 6 μg of MLKL expression vectors for 24 h (HEK293T) or for 12 h then treated with T/S/Z for 8 h (HEK293T-RIP3). The cells were harvested, and the whole-cell lysates were incubated with or without 100 μM compounds on ice for 4 h, then mixed with DMSO (as a control) or 500 μM crosslinker DSG for 30 min. After stopping the reaction, samples were separated by SDS-PAGE and analyzed by western blotting.

### Recombinant protein purification

The cDNAs encoding wild-type or mutant forms of the truncated kinase-like domain (human,179~471 aa) MLKL were subcloned into the expression vector pFastBac-I (Life Technologies, Inc.) with an N-terminal GST tag followed by sequence of HRV3C Protease (Leu-Glu-Val-Leu-Phe-Gln-Gly-Pro) cleavage site. The plasmid was transformed into DH10Bac E. coli cells (Weidi Biotechnology Co, Ltd). The recombinant viral DNA bacmid was purified according to the Bac-To-Bac Baculovirus Expression procedure and confirmed by PCR amplification analysis. The proteins were expressed in Sf9 insect cells and were purified with Glutathione Sepharose 4B (GE Healthcare, cat#17075604) in wash buffer (20 mM Hepes-NaOH pH 7.5, 150 mM NaCl, and 3 mM DTT). The GST tag was removed by on-column digestion with homemade HRV3C Protease for overnight at 4 °C. The proteins were collected and concentrated for further purification by Superdex 75 or Superdex 200 gel filtration chromatography (GE Healthcare, cat# 17-5174-01 and cat# 28-9909-44). Protein was eluted in storage buffer (20 mM Hepes-NaOH pH 7.5 and100 mM NaCl) for storage.

The cDNAs encoding wild-type or mutant forms of MLKL (126 ~181 aa) subcloned into the expression vector PET-SUMO (Life Technologies, Inc.).The plasmid was transformed into BL21(DE3) E. coli cells (Weidi Biotechnology Co, Ltd). The proteins were purified with NTA resin (GE Healthcare), then further purification by Superdex 200 gel filtration chromatography (GE Healthcare, cat#28990944). Protein was eluted in storage buffer (20 mM Hepes-NaOH pH 7.5 and100 mM NaCl) for storage.

### Blue native polyacrylamide gel electrophoresis

Blue Native-PAGE was using Tris-glycine native gel. Recombinant wild-type or mutant forms of MLKL-KLD proteins (2 mg/mL) were mixed with Native-PAGE loading buffer (containing 0.1% Coomassie brilliant blue G250). Analytes were applied to 10% Tris-glycine native gel in blue cathode buffer with 0.02% Coomassie brilliant blue G250, and electrophoresis was performed at room temperature, 115 V, 40 mA. The gels were stained with Coomassie Brilliant Blue G250.

### Cell survival assay

The cells were seeded into 96-well plates and allowed to grow for about 12 h at the 5000 cells/well. Cell survival assay was performed using the CellTiter-Glo Luminescent Cell Viability Assay kit (Promega, cat# G7573) according to the manufacturer’s instructions. Luminescence was recorded with a SpectraMax i3 multimode plate reader from Molecular Devices.

### Immunoblotting

Cells cultured on 60 mm dishes were collected and washed once with DPBS. The harvested cells were resuspended in lysis buffer containing 20 mM Hepes-NaOH pH 7.5, 150 mM NaCl, 1% Triton X-100, 10% glycerol, and complete protease inhibitor (Roche) for 30 min on ice. Then, cell lysates were centrifuged at 15,000 g at 4 °C for 20 min. The soluble fraction was collected and boiled with loading buffer. The samples were subjected to SDS-PAGE and transferred onto nitrocellulose membrane. The membrane was blocked with 5% skim milk for 1 h at room temperature and incubated with indicated antibody. The membrane was visualized using Amersham Imager 680(GE Healthcare, cat# 29270769) with the ECL reagent (PE, cat# NEL105001EA).

### Crystallization and structure determination

Recombinant kinase-like domain mutated MLKL proteins were concentrated to 5 mg/ml and subjected to robotic crystal trials. All crystals were grown at 20 °C using the vapor-diffusion on plates method. Crystals of human MLKL kinase-like domain (phosphor-sites’ mutant T357A/S358A, AA) appeared in a well buffer containing 0.1 M HEPES pH 7.5, 14% w/v PEG6000, and 5% EG. Crystals of human MLKL kinase-like domain (phosphor-mimic mutant T357E/S358D, ED) appeared in a well buffer containing 0.1 M HEPES pH 7.5, 5% v/v (+/−)−2-Methyl-2,4-pentanediol, and 10% w/v Polyethylene glycol 10000. Native datasets were collected at the Shanghai Synchrotron Radiation Facility (SSRF) beamline BL17U1 and BL19U1 were processed with HKL2000 package^22^. Data collection statistics are summarized in Table S1. Phases for MLKL kinase-like domain mutants AA and ED were first obtained by molecular replacement with Phaser using wild-type MLKL kinase-like domain structure (PDB codes: 4M67^6^) as the search model^23^. Phenix was used to build and refine the model^24^.

### PISA analysis

Contact areas and binding energies of the dimer interface of MLKL kinase-like domain were analyzed using the protein interfaces, surfaces, and assemblies (PISA) web server (www.ebi.ac.uk/pdbe/pisa/)^25^.

### Statistical analysis

The data of cell survival assays are carried out with Microsoft Excel (Microsoft). representative of duplicate or triplicate wells with error bars represent mean ± SD, and similar results were obtained from at least three independent experiments.

## Results

### The dimerization of MLKL kinase-like domain

**Fig. 1:**
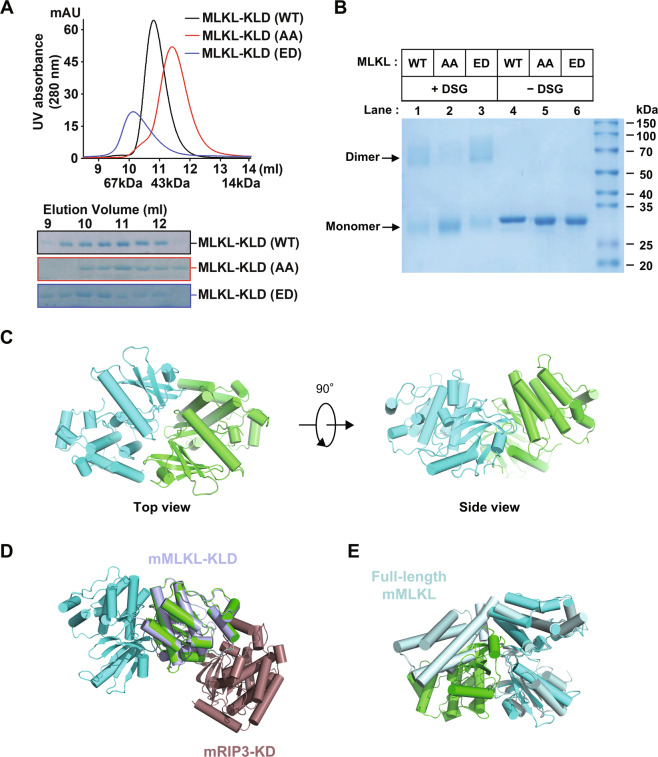
The dimerization of MLKL kinase-like domain. **A** Gel filtration analysis of purified recombinant human MLKL kinase-like domain (KLD) proteins on Superdex 75. The elution fractions were applied to SDS-PAGE followed by Coomassie blue staining. The elution positions of size standards are indicated (BSA, 67 kDa; Ovalbumin, 44 kDa; ribonuclease A, 14 kDa). **B** Detection of self-assembly of recombinant human MLKL kinase-like domain proteins using chemical crosslinker. Aliquots of purified recombinant wild-type or mutant forms of MLKL KLD proteins were mixed with DMSO or crosslinker DSG as described in the MATERIALS AND METHODS, Samples were subjected to SDS-PAGE and stained by Coomassie blue. Abbreviations are as follows: DSG, disuccinimidyl glutarate; WT, wild-type; AA, phosphor-sites’ mutant, T357A/S358A; ED, phosphomimic mutant, T357E/S358D. **C** Two perpendicular views of the overall structure of the phosphomimic mutant (T357E/S358D) of human MLKL kinase-like domain homodimer (green and cyan). All structural figures were prepared with PyMOL. **D** Structural comparison of the phosphomimic mutant (T357E/S358D) of human MLKL kinase-like domain homodimer (green and cyan) with the complex structure of mouse MLKL kinase-like domain (bluewhite) and mouse RIP3 kinase domain (brown) (PDB: 4M69). **E** Structural comparison of the phosphomimic mutant (T357E/S358D) of human MLKL kinase-like domain homodimer (green and cyan) with the full-length structure of mouse MLKL (palecyan, PDB: 4BTF).

The oligomerization of full-length MLKL protein is a critical step of MLKL activation. It is triggered by RIP3-mediated phosphorylation on their kinase-like domain and mediated by self-assembly of the MLKL coiled-coil region. Before RIP3-mediated phosphorylation, the cellular endogenous MLKL proteins remain monomeric. It is still not resolved how phosphorylation in the kinase-like domain of MLKL initiates self-assembly of the coiled-coil domain. To elucidate the mystery of phosphorylation induced kinase-like domain activation, protein behaviors of the recombinant kinase-like domain of wild-type, phosphomimic (T357E/S358D) mutant and phosphor-sites’ mutant (T357A/S358A) of human MLKL proteins were compared by gel filtration chromatography. A peak between the 67 and 14 kDa molecular size markers were observed for wild-type proteins (Fig. 1A, black line). While the behaviors of both T357E/S358D mutant (Fig. 1A, blue line) and T357A/S358A mutant (Fig. 1A, red line) differed from the wild-type. The T357E/S358D mutant protein peak was shown between the 67 and 43 kDa as a dimer (blue line), while the T357A/S358A mutant protein peak was shown between the 43 and 14 kDa as a monomer (red line). It suggested MLKL kinase-like domain intrinsically formed monomer or dimer, and phosphorylation at T357 and S358 will drive a transition from monomer to the dimer. It was confirmed by subjecting the wild-type, T357A/S358A, and T357E/S358D mutants of MLKL to Blue-native-PAGE or SDS-PAGE in the absence and presence of an amine-to-amine crosslinker, disuccinimidyl glutarate (DSG) (Fig. S1A and B). All three proteins including T357A/S358A mutants could run at dimeric size on SDS-PAGE in the presence of DSG (lane 1–3). But clearly, the dimerized proteins of T357A/S358A mutants (lane 2) were less than that of wild-type (lane 1) or T357E/S358D mutants (lane 3).

To reveal the molecular mechanism of MLKL kinase-like domain dimerization, we crystallized the recombinant the phosphor-sites’ mutant (T357A/S358A) and phosphomimic mutant (T357E/S358D) forms of MLKL kinase-like domain proteins (Table S1). Interestingly both the crystal structure of T357E/S358D (PDB code 6LK5) and T357A/S358A (PDB code 6LK6) are similar to the reported structure of wild-type protein (PDB code 4M67), exhibiting a canonical kinase fold, with an N-lobe and a C-lobe (Fig. S1B). The N-lobe comprises an antiparallel, five-stranded β-sheet, and an activation αC helix. The C-lobe contains seven α-helices and a pair of β-strands. Considering of the intrinsic tendency of MLKL kinase-like domain dimerization, we proposed that the high protein concentrations in crystals may promote the formation of a specific crystal lattice that stabilizes the dimer form of MLKL kinase-like domain protein. Examination of the crystal lattice of the kinase-like domain reveals three crystallographic dimers (Fig. S1C–E). The MLKL kinase-like domain is close to the intermediate coiled-coil domain. Therefore, the N-termini of kinase-like domains must be close to each other in oligomerized MLKL. For that reason, we proposed that two of crystallographic dimers were functionally irrational (Fig. S1C and D) because the distance between the two N-termini of these dimers are too long (about 52 and 94 Å between the Cα of the Q192 in these dimers). Only one crystallographic dimer has adjacent N-termini (about 9 Å between the Cα of the Q192) (Fig. 1C, and Fig. S1E). The functional rationality of this dimer was confirmed by comparing the structure of the RIP3-MLKL complex. It showed that MLKL dimer did not interfere the binding with RIP3 (Fig. 1D). Moreover, the dimer form of MLKL kinase-like domain conflicted with the monomeric full-length structure of mouse MLKL by steric hindrance with MLKL N-terminal four-helix buddle and coiled-coil part (Fig. 1E). This structure of full-length mouse MLKL (PDB code: 4BTF) was considered to be autoinhibited. It suggested kinase-like domain dimerization disfavor MLKL auto-inhibitory conformation. As indicated by the formal gel filtration analysis of MLKL phosphomimic mutant (Fig. 1A), the phosphorylation by RIP3 will drive a transition from monomer to a dimer of MLKL kinase-like domain, suggesting the direct function of RIP3-phosphorylation on MLKL is to disrupt the MLKL monomeric conformation by promoting MLKL kinase-like domain dimerization.

### MLKL kinase-like domain dimerization is required for MLKL-dependent necroptosis

**Fig. 2:**
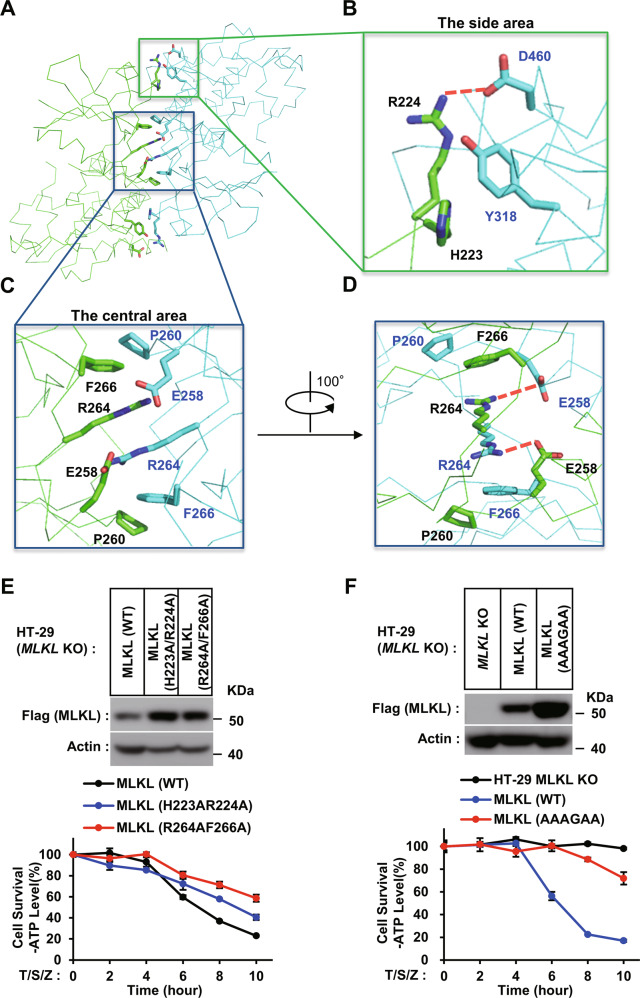
MLKL kinase-like domain dimerization is required for MLKL-dependent necroptosis. **A** Overall structure of MLKL kinase-like domain dimer (green and cyan), illustrating the central and the side of dimer interface. The interface residues involved in intermolecular bonding are shown in sticks. **B** A close-up view on the side of the dimer interface. Arg224 of one MLKL molecular interacted with Glu460 of the other molecular by ionic bond. The phenyl rings from Tyr318 of one MLKL molecular hydrophobic interacted with the hydrocarbon chain of Arg224 and backbone of His223 of the other molecular. Ionic bond is in this and all other figures are represented by red dashed lines. **C**, **D** Close-up views on the center of dimer interface. Glu258 of one MLKL molecule ionically interacted with Arg264 of the other molecule. Pro260 of one MLKL and Phe266 of the other molecular stack against each other by CH/π interaction. **E**, **F** The effect of dimerization mutants of MLKL on necrosis. *MLKL* KO HT-29 cells stably expressing C-terminal flag-tagged wild-type (WT) or dimerization mutants H223A/R224A and R264A/F266A (E) or H223A/R224A/E258A/P260G/R264A/F266A (AAAGAA) (F) of MLKL by lentivirus infection were treated with T/S/Z for the indicated time. The number of surviving cells was determined by measuring ATP levels using the CellTiter-Glo kit (lower panel). The data are presented as the mean ± SD of duplicate wells. Similar results were obtained from at least three independent experiments. Abbreviations are as follows: T, TNF-α; S, Smac mimetic; Z, z-VAD-fmk. The final concentrations of 10 ng/ml TNF-α, 100 nM Smac mimetic, and 20 μM z-VAD-fmk were used. Identical concentrations of these necroptosis-inducing agents were used in subsequent experiments unless otherwise stated. The untreated cells were harvested and whole-cell extracts were prepared and normalized to the same concentration. Aliquots of 20 μg whole-cell lysates were subjected to SDS-PAGE followed by western blot analysis of MLKL(by anti-Flag antibody) and β-Actin which is shown as a loading control (upper panel).

The structure of dimeric MLKL kinase-like domain is symmetric (Fig. 1C). The dimer interface is comprised of the linker region between helix αC(α1) and strand β4, the loop between strands β2 and β3, and some individual residues in helix α3 and α8 (Fig. 2A). Analysis by PISA (Proteins, Interfaces, Structures, and Assemblies) server shows the contact surface area AF is around 1162.2 Å^2^, and 35 residues involved in dimer assembly. The dimer interface was composed by ten hydrogen bonds and eight salt bridges and some of hydrophobic interactions^25^. In the center of dimer interface, Glu258 of one MLKL molecule ionically interacted with Arg264 of the other molecule; Pro260 of one MLKL and Phe266 of the other molecular stack against each other by CH/π interaction. At the side of dimer interface, Arg224 of one MLKL molecular interacted with Glu460 of the other molecular by ionic bond; and the phenyl rings from Tyr318 of one MLKL molecular hydrophobic interacted with hydrocarbon chain of Arg224 and backbone of His223 of the other molecular (Fig. 2B–D). To check the importance of MLKL kinase-like domain dimerization in necroptosis signaling, we constructed MLKL mutants to disrupt the side and/or central area of the dimer interface. The effects of these mutants on necroptosis were checked in MLKL-knockout HT-29 cells. It shows that both mutations (R264A/F266A) destroying the central area and mutations (H223A/R224A) destroying the side area of the dimer interface postponed MLKL dependent necroptosis. The mutations on the central area showed more effectible than mutations on the side area in necroptosis inhibition(Fig. 2E). The six-residues mutations (H223A/R224A/E258A/P260G/R264A/F266A, AAAGAA) destroying both the side and central binding interfaces could block necroptosis efficiently (Fig. 2F).

### MLKL-KLD dimerization initiate the monomer-oligomer transition of MLKL upon RIP3-phosphorylation

**Fig. 3:**
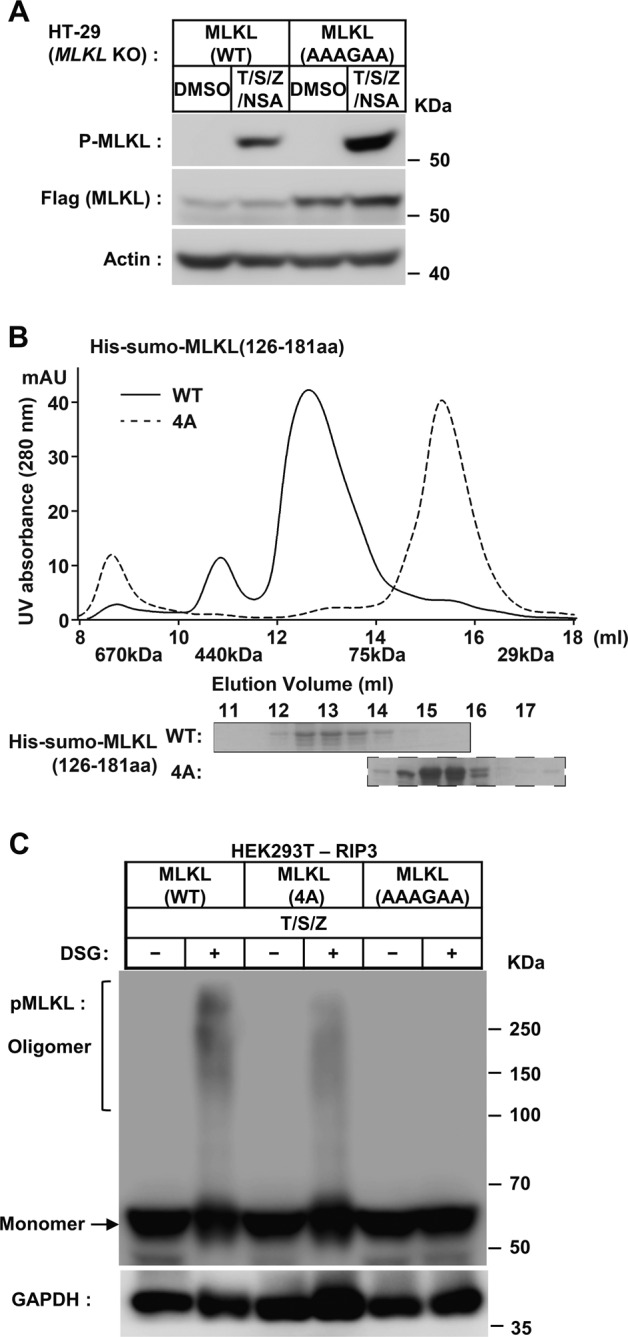
MLKL-KLD dimerization mutant blocks necroptosis by preventing MLKL monomer-oligomer transition. **A** *MLKL* KO HT-29 cells stably expressing flag-tagged wild-type (WT) or dimerization mutant (AAAGAA) of MLKL were treated with DMSO or T/S/Z combine with MLKL inhibitor 2 μM Necrosulfonamide (NSA) for 12 h. Then, the cells were harvested and whole-cell lysates were prepared and subjected to SDS-PAGE followed by western blot analysis using anti-phosphor-MLKL and anti-Flag antibodies. The expression of β-Actin was shown as a loading control. **B** Gel filtration analysis of purified recombinant proteins of His-Sumo tagged human MLKL (126~181 aa) containing the internal coiled-coil region on Superdex 200. The elution fractions were applied to SDS-PAGE followed by Coomassie blue staining. The elution positions of size standards are indicated (Thymoglobulin, 670 kDa; Ferritin, 440 kDa; Aldolase, 75 kDa; Carbonic Anhydrase, 29 kDa). **C** Plasmids containing Wild-type (WT), KLD-dimer mutant (AAAGAA), or coiled-coil mutant(4 A) were transfected transiently into HEK293T cells with stable-expressed human RIPK3 (HEK293T-RIPK3) for about 12 h in 60 mm dishes. Then the cells were treated with T/S/Z for 8 h and harvested. And the whole-cell lysates were mixed with DMSO or crosslinker DSG as described in the MATERIALS AND METHODS. The samples were analyzed by western blotting using an anti-Flag (MLKL).

The MLKL(AAAGAA) mutations on KLD dimer interfaces inhibited TNF-induced necroptosis. To avoid the possibility that these mutations affect the proper folding of MLKL to disrupt MLKL function, we checked the phosphorylation of MLKL(AAAGAA) mutant by RIP3. It showed that the p-MLKL signals were comparable between wild-type and a mutant form of MLKL, suggesting that the cellular MLKL mutant was folded well to be the substrate of RIP3 as wild-type MLKL (Fig. 3A). The dimerization of MLKL KLD was considered as the direct consequence of RIP3-phosphorylation, so that monomer-oligomer transition of MLKL was the downstream step and could be prevented by mutations on KLD dimer interface. It has been reported that the MLKL contained two coiled-coil regions, the N-terminal one is in the MLKL 4HB region, and the internal one is in the brace region linked the 4HB and KLD of MLKL^13^. We found the recombinant proteins of internal coiled-coil in brace region (MLKL_126~181 aa) alone could form oligomer in vitro, while the mutations on the four critical residues to alanine (L162AL165AM169AI172A, 4 A) prevented their oligomerization in gel-filtration analysis (Fig. 3B and S2A). It was confirmed by treatment of wild-type and coiled-coil mutant (4 A) forms of MLKL proteins (126~181 aa) with chemical crosslinker DSG (Fig. S2B). It showed that although, both coiled-coil mutant (4 A) and KLD dimerization mutant (AAAGAA) of MLKL were not sensitive to necroptosis induction (Fig. S2C), they behaved differently on necroptosis-dependent monomer-oligomer transition. The 4 A mutations could only decrease the MLKL oligomerization upon necroptosis induction, while the KLD dimer (AAAGAA) mutations blocked monomer-oligomer transition in the same condition (Fig. 3C). This confirmed that KLD dimerization was the upstream step.

### Kinase-like domain dimerization is the common step of RIP3-induced MLKL activation in different species

**Fig. 4:**
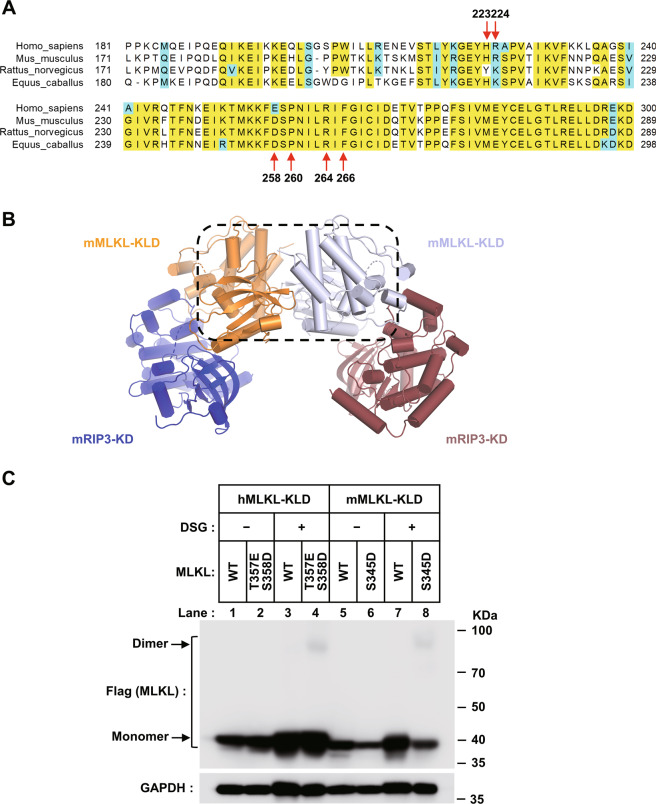
Kinase-like domain dimerization is a conserved step of RIP3-induced MLKL activation among mammalian species. **A** Sequences alignment of MLKL orthologs in four vertebrate species. The identical and conserved residues were highlighted in yellow and cyan respectively. Dimer interface residues of human MLKL showed in Fig. 2 are indicated by red arrows. **B** The overall structure of the dimeric mouse RIP3-MLKL complex (RIP3: blue or brown and MLKL: carrot or silver). The dotted box indicates the interface of the mouse MLKL kinase-like domain dimer. **C** Phosphomimic mutation promotes kinase-like domain dimerization in both human and mouse MLKL. Flag-tagged wild-types and phosphomimic mutants (S345D on mouse MLKL or S357ES358D on human MLKL) MLKL kinase-like domain proteins were exogenously expressed in HEK293T cells. Then whole-cell lysates were mixed with DMSO or crosslinker DSG as described in the MATERIALS AND METHODS. The samples were analyzed by western blotting using an anti-Flag antibody.

It has been reported that the crystal structures of human (PDB code: 4MWI), rat (PDB code: 6VBZ), and horse (PDB code: 6VC0) MLKL kinase-like domain presented a closed-state conformation, charactered by a conserved slat-bridge salt bridge (K230–E250 in human, K219–E239 in rat and K228–E248 in horse) which is equivalent of the K72–E91 interaction in protein kinase A(PKA). While the mouse MLKL kinase-like domain presents a more open-state conformation (from PDB 4BTF), charactered by a unique salt bridge of K219–Q343^5,26,27^. Therefore, the presumption of divergent activation mechanisms among these mammalian MLKL orthologues was raised^26^. The species-divergent model may suggest the KLD dimerization is conserved step of MLKL activation in human, rat, and horse, but not in mouse. Indeed, similar MLKL dimer could be found in the crystal lattice of rat and horse crystal structure (Fig. S3A and B). But interestingly, the residues in human MLKL dimer interfaces mediating intermolecular interactions are conserved not only in human, rat, and horse but also in mouse MLKL (Fig. 4A). Although similar KLD dimer could not find from the crystal lattice of full-length mouse MLKL structure (PDB code: 4BTF) or mouse MLKL KLD structure (PDB code: 4M68), it could be found from the crystal lattice of mouse RIP3-MLKL complex structure (PDB code: 4M69) (Fig. 4B). So that, kinase-like domain dimerization is also a conserved activation step of mouse MLKL. To prove that, we constructed wild-types, phosphomimic mutants (mouse S345D and human T357ES358D) of MLKL kinase-like domain proteins for exogenous expression in HEK293 cells. In the presence of chemical crosslinker DSG, both of phosphomimic mutants of human and mouse MLKL (T357ES358D and S345D) showed more signals at dimer position than wild-type MLKL (Fig. 4C). It confirmed mouse MLKL kinase-like domain dimerization is also the direct consequence caused by phosphorylation at serine 345 by RIP3. Therefore, KLD dimerization is the common step of RIP3-induced MLKL activation in different mammalian species.

## Discussion

**Fig. 5:**
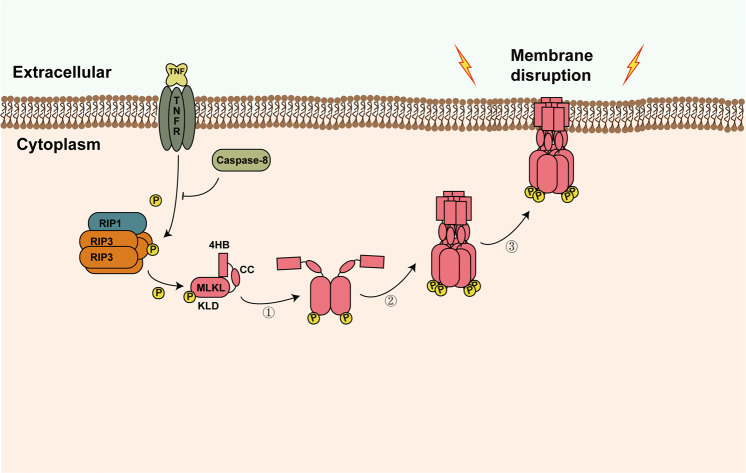
Schematic model of MLKL mediated necroptosis execution. The serial activation steps of RIP3-phosphorylated MLKL during necroptosis execution are indicated: Step ①, phosphorylation by RIP3 induce MLKL kinase-like domain dimerization; Step ②, The self-interaction of kinase-like domain will initiate the self-assembly of the internal coiled-coil region, which causes MLKL oligomerization; Step ③, oligomerized MLKL translocated to the plasma membrane to cause membrane disruption.

Phosphorylation of MLKL by RIP3 is a hallmark of necroptosis, which causes MLKL activation and initiates MLKL dependent necroptosis execution. Here, we found MLKL kinase-like domain dimerization is a critical step of MLKL activation and necroptosis, which is conserved in mammalian species. MLKL kinase-like domain dimerization interrupts the monomeric conformation and induces oligomerization of full-length MLKL, suggesting a serial activation model of MLKL mediated necroptosis execution (Fig. 5): Step 1, phosphorylation by RIP3 induce MLKL kinase-like domain dimerization; Step 2, The self-interaction of kinase-like domain will initiate the self-assembly of the internal coiled-coil region; Step 3, oligomerized MLKL translocated to the plasma membrane to cause membrane disruption and cell necrosis. This model suggests MLKL activation is a sequential process and dimerization is direct consequence of RIP3-mediated phosphorylation in the MLKL kinase-like domain and initiate MLKL oligomerization. As a supporting evidence, mutations on MLKL internal coiled-coil region disrupting CC-dependent MLKL self-assembly could completely block necroptosis, but only attenuated MLKL oligomerization. While the mutations disrupting MLKL KLD dimerization could completely block both MLKL oligomerization and necroptosis (Figs. 2F, 3C and S2C)

Divergent activation mechanisms among mammalian MLKL orthologues have been raised based on the structural differences between human and mouse MLKL kinase-like domain of human and^20,26,27^. But our results indicated these differences may not affected MLKL activation process after RIP3-phosphorylation. All the mammalian MLKL uses kinase-like domain dimerization as the general step next to RIP3-phoshorylation which is critical to commit MLKL activation process. Besides that, the MLKL KLD dimer is only found in the crystal lattice of the mouse RIP3-MLKL complex (PDB code: 4M69), but not in crystal lattices of mouse MLKL full-length and KLD structure (PDB code: 4BTF and 4M68). It suggested the MLKL KLD conformations in 4BTF and 4M68 is in different state from MLKL in the RIP3-MLKL complex. The mouse MLKL KLD in RIP3-MLKL complex or the reported human, rat, and horse MLKL KLD structure are probably in an active-state. While the MLKL KLD conformation in 4BTF and 4M68 structure may be in a closed or inactive state. Interestingly, the 4BTF and 4M68 structure have a unique K213–Q343 slat-bridge which stabilized their closed-state conformations. Mutants changing the sites of K219/Q343 in mouse MLKL (or conserved sites K230/Q356 in human MLKL) induce MLKL activation which causes RIP3-independent necroptosis^5,14,18^. It indicates the human, rat, or horse MLKL may also have similar closed-state conformation hallmarked with the K230–Q356 salt-bridge in human or other conserved salt-bridges in other species, which will be captured in the future by trails of more crystallization conditions. Indeed, two new monobody-stabilized human KLD structures have been recently reported which showed different conformations^28^. The monobody-27 binding MLKL KLD (PDB code: 7JW7) shows active-state conformation, which contains the functional dimer interface in the crystal lattices. While the monobody-33 binding MLKL KLD (PDB code: 7JXU) shows closed-state conformation hallmarked with the K230–Q356 salt-bridge. And the functional dimer interface we identified was not in the monobody-33 binding MLKL crystal lattices. These clearly indicates the previous MLKL specie-divergent activation model which is based on the structural differences of mouse MLKL KLD from MLKL of other species need to be optimized and amended^26^. Importantly, in all active-state MLKL KLD structures, residues around the RIP3-target residues S345 in mouse (or T357/S358 in human) are disordered. While the mouse K219–Q343 (or human K230–Q356) salt-bridge stabilized KLD structure (PDB code: 4BTF, 4M68, and 7JXU) the nearby residues around RIP3-target mouse S345 (or human T357/S358) form helix conformations. It may suggest the phosphorylation on mouse S345 (or human T357/S358) will disturb the helix conformation to a disordered state which breaks mouse K219–Q343 (or human K230–Q356) salt-bridge (indicated by the close proximity of mouse Q343 or human Q356 with the mouse S345 or human T357/S358 in MLKL) to drive the transition of MLKL KLD proteins from inactive-state to active dimers.

## Supplementary information

Supplemental Figure 1~3 with legends

Table-S1

## Data Availability

Atomic coordinates for the T357ES358D and T357AS358A mutant forms of human MLKL kinase-like domain have been deposited in the Protein Data Bank with the accession numbers PDB 6LK5 and PDB 6LK6, respectively. Other data are available from the corresponding authors upon request.
