# Supplementary figures and images for: The MLKL kinase-like domain dimerization is an indispensable step of mammalian MLKL activation in necroptosis signaling
Source: Cell Death Dis. 2021 Jun 22;12(7):638. doi: 10.1038/s41419-021-03859-6 (PMC8219780; doi:10.1038/s41419-021-03859-6)

Figure S1

A

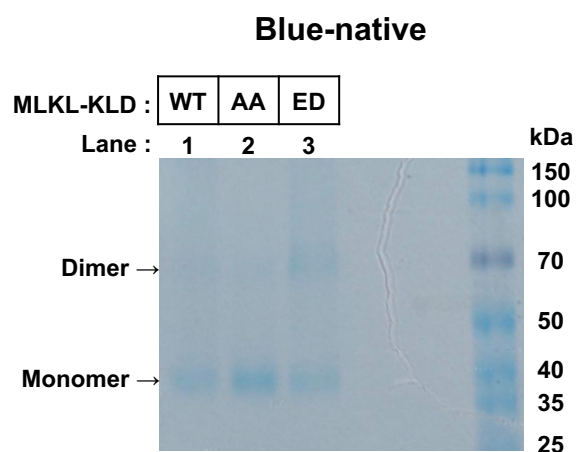

B

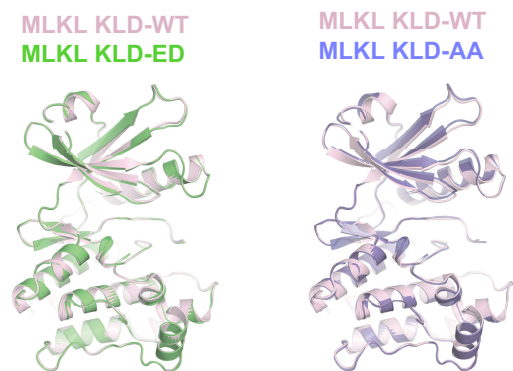

C

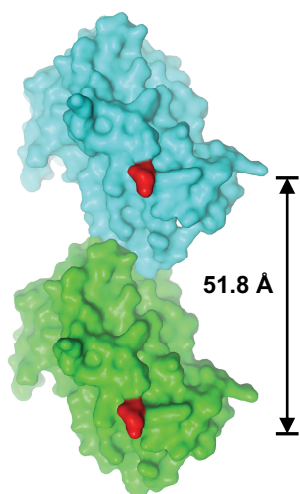

D

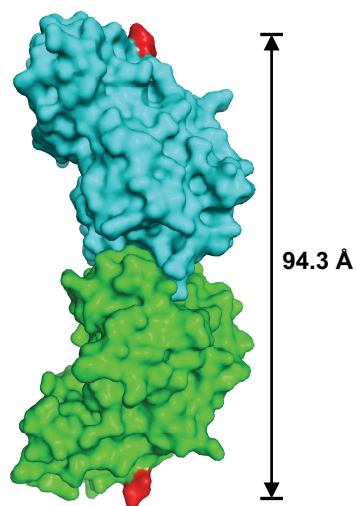

E

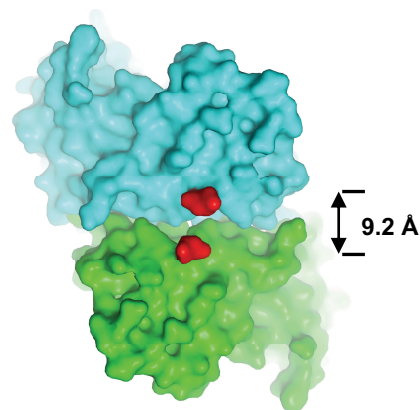

Figure S2

A

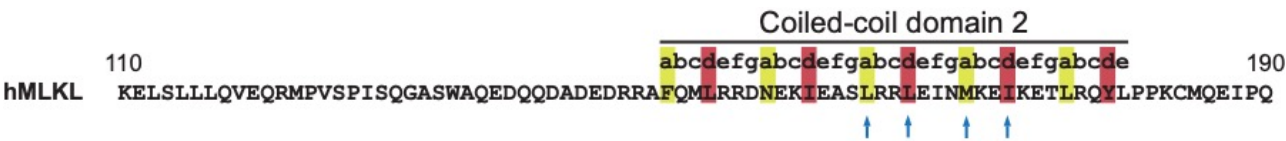

B

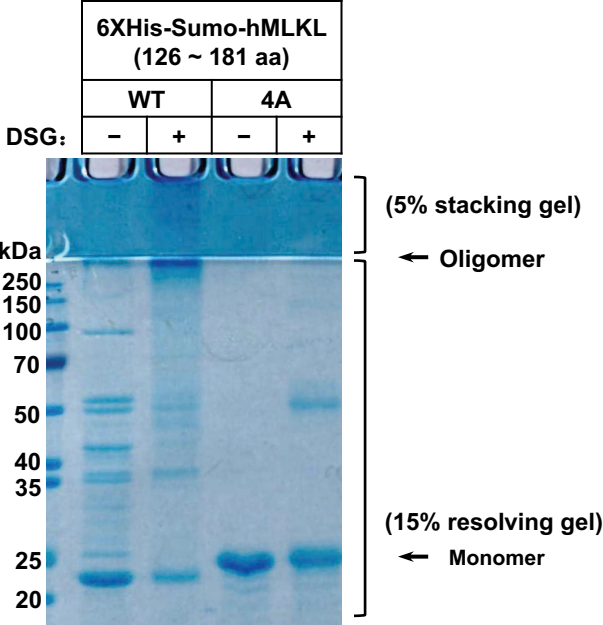

C

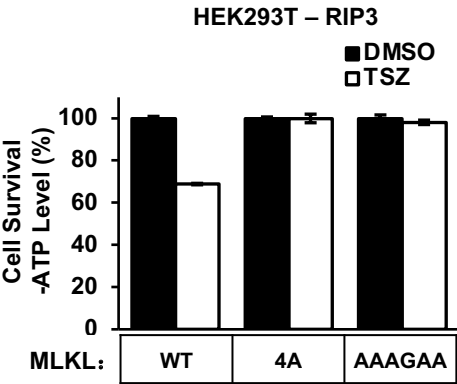

Figure S3

**A**

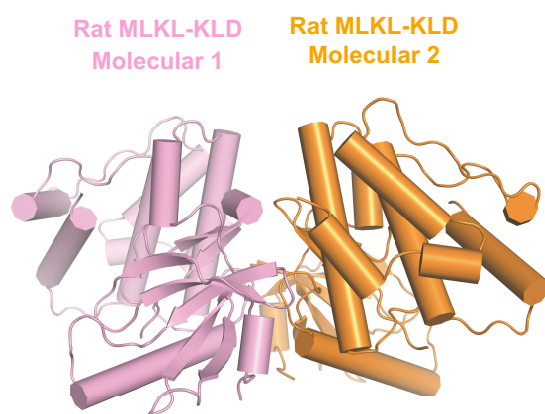

**B**

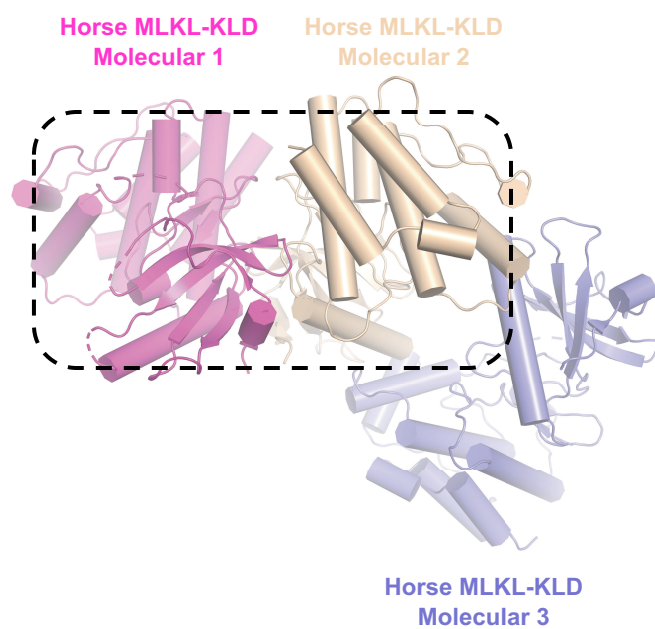

Supplement: Supplementary file 1 — Supplemental Figure 1~3 with legends [file 41419_2021_3859_MOESM1_ESM.pdf]
