# Supplementary material for: The MLKL kinase-like domain dimerization is an indispensable step of mammalian MLKL activation in necroptosis signaling
Source: Cell Death Dis. 2021 Jun 22;12(7):638. doi: 10.1038/s41419-021-03859-6 (PMC8219780; doi:10.1038/s41419-021-03859-6)
Supplement: Supplementary file 2 — Table-S1 [file 41419_2021_3859_MOESM2_ESM.docx]

Supplementary Table 1. X-ray crystallography data collection and refinement statistics

|  | Human MLKL(179-471)  (T357ES358D) | Human MLKL(179-471)  (T357AS358A) |
| --- | --- | --- |
| Data collection |  |  |
| Space Group | C 2 2 2_1_ | C 2 2 2_1_ |
| Cell dimensions |  |  |
| *a, b, c* (Å) | 71.74 74.85 127.39 | 70.82, 75.71, 128.49 |
| 𝛼, β, 𝛾 (°) | 90, 90, 90 | 90, 90, 90 |
| Resolution(Å) | 50~2.50 (2.57~2.50) | 50～2.41 (2.47～2.40） |
| R-merge | 0.15 | 0.16 |
| *I*/ 𝜎*I* | 20.46 (2.51) | 3.33(2.42) |
| Completeness (%) | 99.9 (99.9) | 68.7(94.5) |
| Redundancy | 12.7 (11.9) | 5.5 (1.1) |
| Refinement |  |  |
| Reflections used in refinement | 11569 (848) | 12364 (665) |
| R-work | 0.194 | 0.209 |
| R-free | 0.238 | 0.247 |
| No. non-hydrogen atoms  Protein  Ligand  Water | 2184  2138  0  46 | 2158  2138  0  20 |
| Average B, all atoms (Å^2^) | 56.0 | 62.0 |
| R.M.S deviations  Bond lengths (Å)  Bond angles (°) | 0.004  1.256 | 0.005  1.247 |

Statistics for the highest-resolution shell are shown in parentheses.
